# Choline Rescues Behavioural Deficits in a Mouse Model of Rett Syndrome by Modulating Neuronal Plasticity

**DOI:** 10.1007/s12035-018-1345-9

**Published:** 2018-09-15

**Authors:** Eunice W. M. Chin, Wee Meng Lim, Dongliang Ma, Francisco J. Rosales, Eyleen L. K. Goh

**Affiliations:** 1 Neuroscience Academic Clinical Programme, Duke-NUS Medical School, 20 College Road, Singapore, 169856 Singapore; 2 Department of Research, National Neuroscience Institute, Singapore, 308433 Singapore; 3 NUS Graduate School for Integrative Sciences and Engineering, National University of Singapore, Singapore, 117456 Singapore; 4 Abbott Nutrition Research and Development, Columbus, OH USA; 5 Department of Physiology, Yong Loo Lin School of Medicine, National University of Singapore, Singapore, 117597 Singapore; 6 KK Research Center, KK Women’s and Children’s Hospital, Singapore, 229899 Singapore

**Keywords:** Rett syndrome, Choline, Synaptic plasticity, Nutrition, Neurodevelopment

## Abstract

Rett syndrome (RTT) is a postnatal neurodevelopmental disorder that primarily affects girls, with 95% of RTT cases resulting from mutations in the methyl-CpG-binding protein 2 (*MECP2*) gene. Choline, a dietary micronutrient found in most foods, has been shown to be important for brain development and function. However, the exact effects and mechanisms are still unknown. We found that 13 mg/day (1.7 × required daily intake) of postnatal choline treatment to *Mecp2*-conditional knockout mice rescued not only deficits in motor coordination, but also their anxiety-like behaviour and reduced social preference. Cortical neurons in the brains of *Mecp2*-conditional knockout mice supplemented with choline showed enhanced neuronal morphology and increased density of dendritic spines. Modelling RTT in vitro by knocking down the expression of the MeCP2 protein with shRNA, we found that choline supplementation to MeCP2-knockdown neurons increased their soma sizes and the complexity of their dendritic arbors. Rescue of the morphological defects could lead to enhanced neurotransmission, as suggested by an observed trend of increased expression of synaptic proteins and restored miniature excitatory postsynaptic current frequency in choline-supplemented MeCP2-knockdown neurons. Through the use of specific inhibitors targeting each of the known physiological pathways of choline, synthesis of phosphatidylcholine from choline was found to be essential in bringing about the changes seen in the choline-supplemented MeCP2-knockdown neurons. Taken together, these data reveal a role of choline in modulating neuronal plasticity, possibly leading to behavioural changes, and hence, a potential for using choline to treat RTT.

## Introduction

Rett syndrome (RTT) (OMIM 312750) is a postnatal neurodevelopmental disorder. It is the second leading cause of mental retardation in girls, with an incidence rate of approximately one in 10,000 [1–3]. The disorder manifests almost exclusively in females, with observed lethality in hemizygous males [1, 4–7]. Mutations in the X-linked methyl-CpG-binding protein 2 (*MECP2*) gene were found to be responsible for more than 95% of RTT cases [8–10]. Patients with classical RTT develop seemingly normally until around 18 months of age, where they start to exhibit developmental stagnation. There is general growth deceleration, with microcephaly, weight loss, and muscle hypotonia, resulting in gait and posture dyspraxia. There are also deficits in cognitive and motor skills, characterized by loss of any acquired language and purposeful hand use [11]. Autistic features manifest in RTT patients, such as lack of affect, increased anxiety, and unresponsiveness to social and environmental cues [12].

There is currently no known cure for RTT. Given that the underlying neuronal framework is still relatively intact [13, 14], drug-like compounds [15–17], and nutritional supplementation [18–20] have been used in attempts to augment the functionality of the neurons, and thereby alleviate symptoms of the disorder. Despite the potential, the translation of treatments from animals to humans has not been successful [17, 21]. There is, thus, a need to explore alternative means for the treatment of RTT.

Over the last two decades, there has been a growing interest in the use of nutritional compounds for the treatment of various ailments. Promisingly, supplementation of choline, a common dietary micronutrient, to various mouse models of RTT markedly improved their motoric function [18, 19]. But, the exact mechanism of how choline leads to improved function has not been comprehensively dissociated. This can have important ramifications on finding novel therapeutic strategies for RTT. Thus, we sought to examine the effects of choline on the various functional aspects of RTT, and to elucidate a possible mechanism of action of choline.

## Results

### Choline Supplementation to *Mecp2*-conditional Knockout Animals Rescues Their Behavioural Deficits

**Fig. 1:**
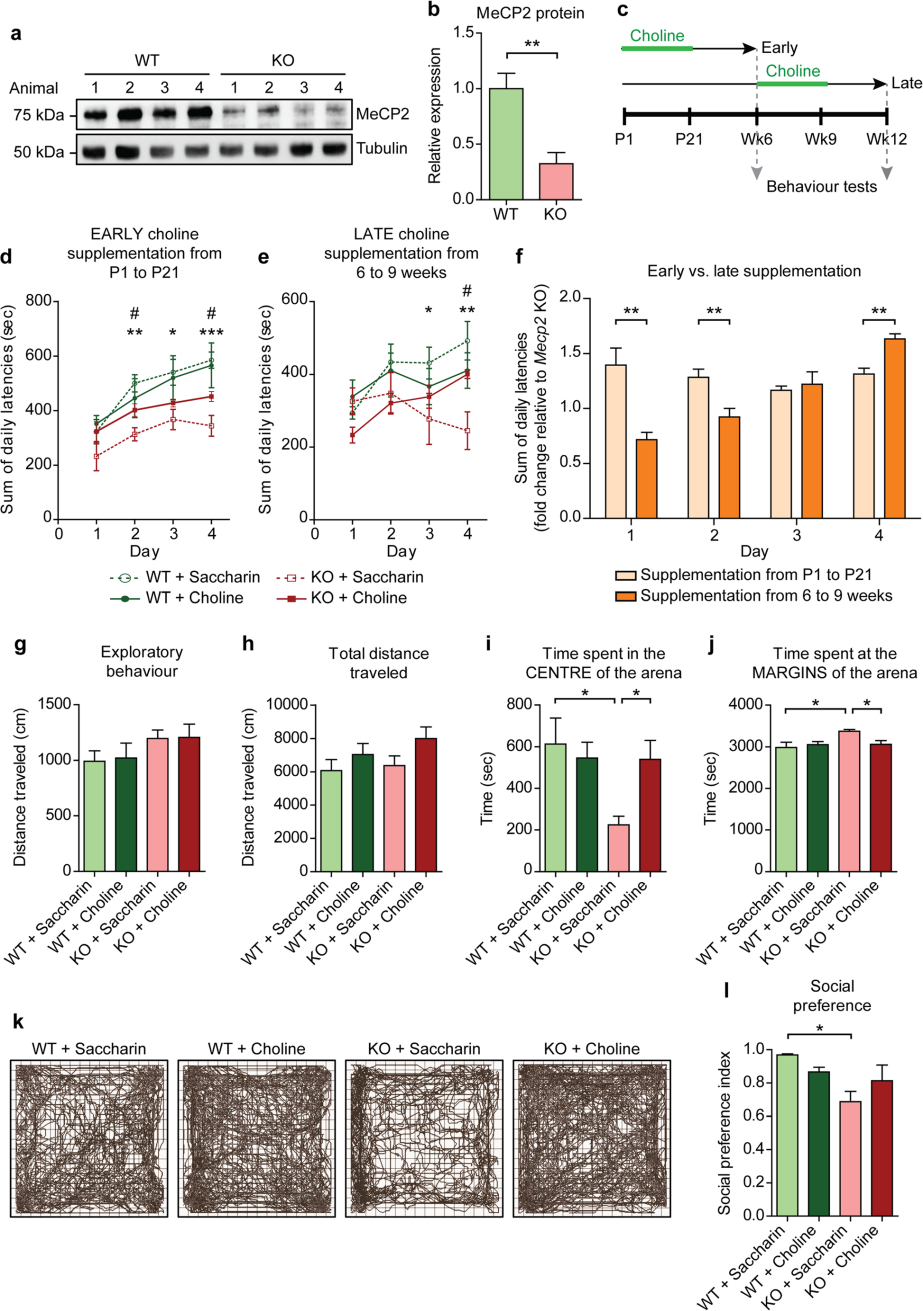
Choline ameliorates behavioural deficits in a mouse model of RTT **a** Representative Western blots showing MeCP2 expression in cortical tissue lysates from wild type (WT) and *Mecp2*-conditional knockout (KO) mice. **b** Quantification of MeCP2 protein expression from **a**. *n* = 4 animals. Student’s *t* test. **c** Schedule of choline treatment and behavioural testing. **d** Mean sum of latencies to fall off an accelerating rotarod from all four trials on each test day by mice supplemented early from P1 to P21 with either vehicle or choline. Asterisk represents comparison between WT + Saccharin and KO + Saccharin, while number sign represents comparison between KO + Saccharin and KO + Choline. **e** Mean sum of latencies to fall from all four trials on each test day by mice supplemented late from 6 to 9 weeks old with either vehicle or choline. **f** Comparison of the improvement in the rotarod test relative to vehicle-supplemented KO mice between mice supplemented early and those supplemented late with choline. **g** Exploratory behaviour of the vehicle- or choline-supplemented WT and KO animals as measured by the distance traveled in the open field test in the first 5 min. **h** Total distance traveled by the animals in the open field test. **i** and **j** Amount of time spent by the animals at the centre **i** and at the margins **j** of the open field arena. **k** Representative traces of the vehicle- or choline-supplemented WT and KO animals in the open field test. **l** The social preference index is given by the ratio of the amount of time that the subject animal spent interacting with a stranger animal to the total amount of time that the subject spent interacting with both the stranger and an inanimate object. *n* = 10–13 animals for each condition. All values are presented as mean ± s.e.m. *p < 0.05, ** *p* < 0.01 and *** *p* < 0.001. One-way ANOVA with Bonferroni post-hoc

To investigate the effects of choline supplementation in a behaving animal, we crossed *Mecp2*-floxed mice with a line expressing Cre recombinase under the *CaMKIIα* promoter. This generated animals with the *Mecp2* gene deleted specifically in the excitatory neurons of the forebrain. This model was chosen as previous studies have shown that the selective loss of MeCP2 in the forebrain was sufficient for the recapitulation of many of the behavioural phenotypes of RTT [22, 23]. In addition, others have demonstrated the importance of the MeCP2 protein in post-mitotic and glutamatergic neurons in contributing to the RTT phenotype [24, 25]. Via Western blotting, we observed a 68% decrease in the expression of MeCP2 protein in the cortices of the *Mecp2*-conditional knockout (KO) animals compared to their wild type (WT) littermates (Fig. 1a, b).

The animals were supplemented with choline in their drinking water from postnatal day 1 (P1) to P21. Approximately 3 weeks after the supplementation schedule, the animals were subjected to a series of behavioural assays (Fig. 1c). *Mecp2*-conditional knockout mice tested at 6 weeks old had significantly lower latencies to fall off the rotarod compared to their WT littermates (Fig. 1d). With choline supplementation from P1 to P21, the performance of the KO animals was rescued. Choline supplemented-KO animals had significantly higher latencies to fall off the rotarod than their vehicle-supplemented counterparts, demonstrating that they had improved motor coordination (Fig. 1d). These results are congruent with previous findings [18, 19]. To examine if the timing of supplementation affected the improvement in motor function, a separate group of animals was given choline when they were between 6 to 9 weeks old, and then tested when they were 12 weeks old (Fig. 1c). Motor coordination was also restored in the later-supplemented KO animals (Fig. 1e). Therefore, choline supplementation is able to rescue the deficit in motor coordination even when administered at a later time point, and that with training, the motor coordination of the later-supplemented KO animals improved as much as that of the early-supplemented ones (Fig. 1f). This suggests that choline has the potential to alleviate RTT symptoms if administered at any one time point.

Although choline supplementation improved motor coordination in the KO animals, we did not observe alterations in their general locomotor activity (total distance traveled) (Fig. 1g) or exploratory behaviour (Fig. 1h) in the open field test. The *Mecp2*-condtional knockout mice spent significantly less time in the centre of the arena and more time at the margins compared to their WT littermates, indicative of anxiety-like behaviour (Fig. 1i, j, k). With choline supplementation, the KO animals spent significantly more time in the centre of the arena and less time at the margins compared to their vehicle-supplemented counterparts (Fig. 1i, j, k). These results suggest that supplementing choline to the *Mecp2*-conditional knockout animals seems to alleviate their anxiety-like phenotype.

Lastly, the KO animals had a significantly lower social preference index than their WT counterparts, suggestive of deficits in their social preference (Fig. 1l). Choline supplemented-KO animals had a higher social preference index than their vehicle-supplemented counterparts (Fig. 1l). Hence, the data suggest that choline supplementation is able to improve preference for social stimuli in the *Mecp2*-conditional knockout animals.

### Choline Supplementation Rescues Neuronal Morphology and Dendritic Spine Density In vivo

**Fig. 2:**
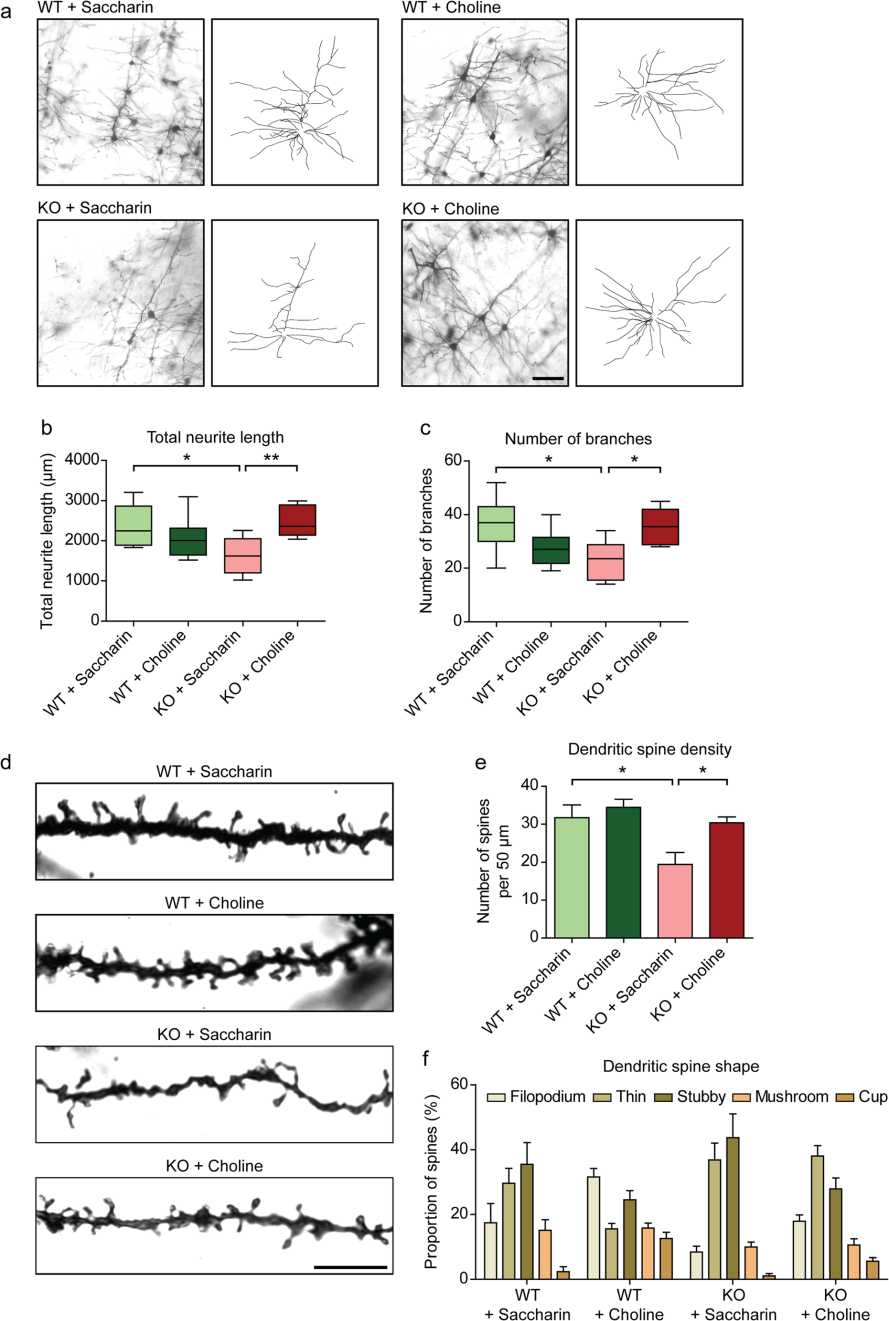
Rescue of neuronal morphology and dendritic spine density in vivo by choline supplementation to *Mecp2*-conditonal knockout mice **a** Representative images and tracings of Golgi-stained neurons from WT and KO mice with vehicle or choline supplementation. Scale bar represents 100 μm. **b** and **c** Quantification of the total neurite length **b** and number of branches **c** of the neurons in the cortices of WT and KO mice supplemented with either vehicle or choline. *n* = 3 animals. **d** Representative images of Golgi-stained neurites from WT and KO mice with vehicle or choline supplementation. Scale bar represents 10 μm. **e** Quantification of the number of dendritic spines per 50 μm length of neurite. **f** Dendritic spines were classified according to their shapes and expressed as a proportion of the total number of spines per 50 μm length of neurite. *n* = 3 animals. All values are presented as mean ± s.e.m. * *p* < 0.05 and ** *p* < 0.01. One-way ANOVA with Bonferroni post-hoc

The improvements in behaviour of the RTT mouse model with choline supplementation point to changes in the underlying neuronal network of the brain. To verify this, Golgi staining was done to examine neuronal morphology. Neurons with significantly shorter neurites were observed in the cortices of KO animals (Fig. 2a, b). In addition, the neurons of the KO animals had less branches than their WT littermates (Fig. 2a, c). These results are congruent with the diminished dendritic trees seen in post-mortem RTT brains [26–28]. Interestingly, choline supplementation to the KO animals rescued their total neurite length (Fig. 2a, b). Furthermore, the neurons had significantly more branching in the choline-supplemented than the vehicle-supplemented KO mice (Fig. 2a, c). Therefore, the rescue of RTT behaviour by choline supplementation could be due to the alterations in the neuronal network.

The number and shape of dendritic spines have generally been used to denote a neuron’s maturity and functionality [29, 30]. The *Mecp2*-conditional knockout animals had significantly less dendritic spines per unit length of neurite than WT animals (Fig. 2d, e). With choline supplementation, the density of dendritic spines in the KO animals was significantly higher than in those without choline treatment (Fig. 2d, e). The KO mice had more stubby and thin spines, and fewer filopodium, mushroom- and cup-shaped spines than the WT mice (Fig. 2f). Choline supplementation to the KO mice increased the proportion of filopodium- and cup-shaped spines, and decreased the proportion of stubby spines, as compared to their vehicle-supplemented counterparts (Fig. 2f). Taken together, these data suggest that choline supplementation to *Mecp2*-conditional knockout animals has the potential to modulate neuronal communication via regulating dendritic spine number and shape.

### Choline Supplementation Rescues Soma Size, Dendrite Length and Branching, and Overall Dendritic Complexity in MeCP2-Knockdown Cortical Neurons

**Fig. 3:**
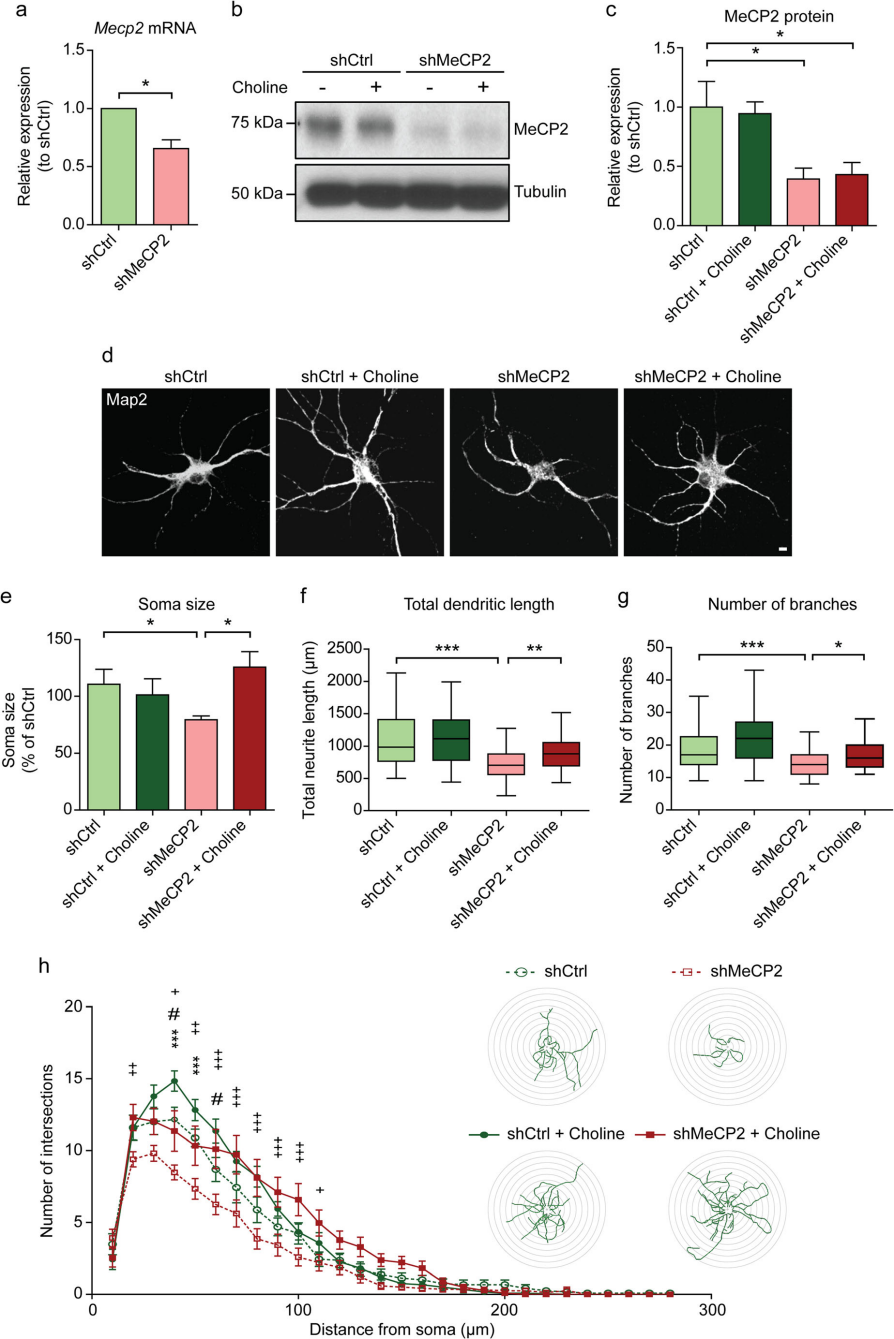
Rescue of neuronal morphology in primary cortical neurons in vitro by choline supplementation **a** Relative expression of *Mecp2* mRNA in cortical neurons transduced with shCtrl or shMeCP2 lentivirus. *n* = 4 cultures. Wilcoxon signed-rank test. **b** Representative Western blots showing MeCP2 expression in DIV 14 cortical neurons with (+) or without (−) choline supplementation. **c** Quantification of MeCP2 protein expression from **b**. mRNA and protein expression levels were normalized to their respective loading controls and expressed relative to shCtrl levels. *n* = 3 cultures. One-way ANOVA with Bonferroni post-hoc. **d** Representative images of DIV 14 cortical neurons immuno-stained for the neuronal marker Map2. Scale bar represents 20 μm. **e** Quantification of soma size of shCtrl and shMeCP2 neurons with or without choline supplementation, expressed as a percentage of shCtrl levels. **f** and **g** Quantification of total dendritic length **f** and number of dendritic branches **g** of shCtrl and shMeCP2 neurons with or without choline supplementation. *n* = 3 cultures. **h** Sholl plot comparing the dendritic arbors of shCtrl and shMeCP2 neurons with or without choline supplementation. The x-axis represents distance from the cell body, while the y-axis represents the number of intersections of a tracing with a particular Sholl shell. The insets show representative tracings of neurons superimposed on concentric circles denoting Sholl shells. Asterisk denotes comparison between shCtrl and shMeCP2; number sign denotes comparison between shCtrl and shCtrl + Choline; and plus sign denotes comparison between shMeCP2 and shMeCP2 + Choline. *n* = 30 neurons per condition. All values are presented as mean ± s.e.m. * *p* < 0.05, ** p < 0.01 and *** *p* < 0.001. One-way ANOVA with Bonferroni post-hoc

To better characterize the morphological changes induced by choline supplementation to the neurons, we modelled RTT in vitro by knocking down MeCP2 in neuronal cultures via lentiviral transduction. Levels of MeCP2 were decreased, by approximately 34.4% and 60.8% respectively for RNA and protein, in the MeCP2-knockdown (shMeCP2) neurons compared to the control (Fig. 3a, b, c).

shMeCP2 neurons had smaller cell bodies and diminished dendritic arbors, with shorter and fewer dendritic branches than shCtrl neurons (Fig. 3d, e, f, g, h). These observations mimic those seen in the RTT brain [26–28]. With choline supplementation, the soma sizes of shMeCP2 neurons increased (Fig. 3d, e). shMeCP2 neurons supplemented with choline had longer and more dendritic branches, with restored overall dendritic complexity, compared to their non-supplemented counterparts (Fig. 3d, f, g, h). These results further elaborate on the potential that choline has in alleviating RTT deficits by rescuing neuronal morphology.

### Choline Supplementation Ameliorates Synaptic Defects in MeCP2-knockdown Neurons

**Fig. 4:**
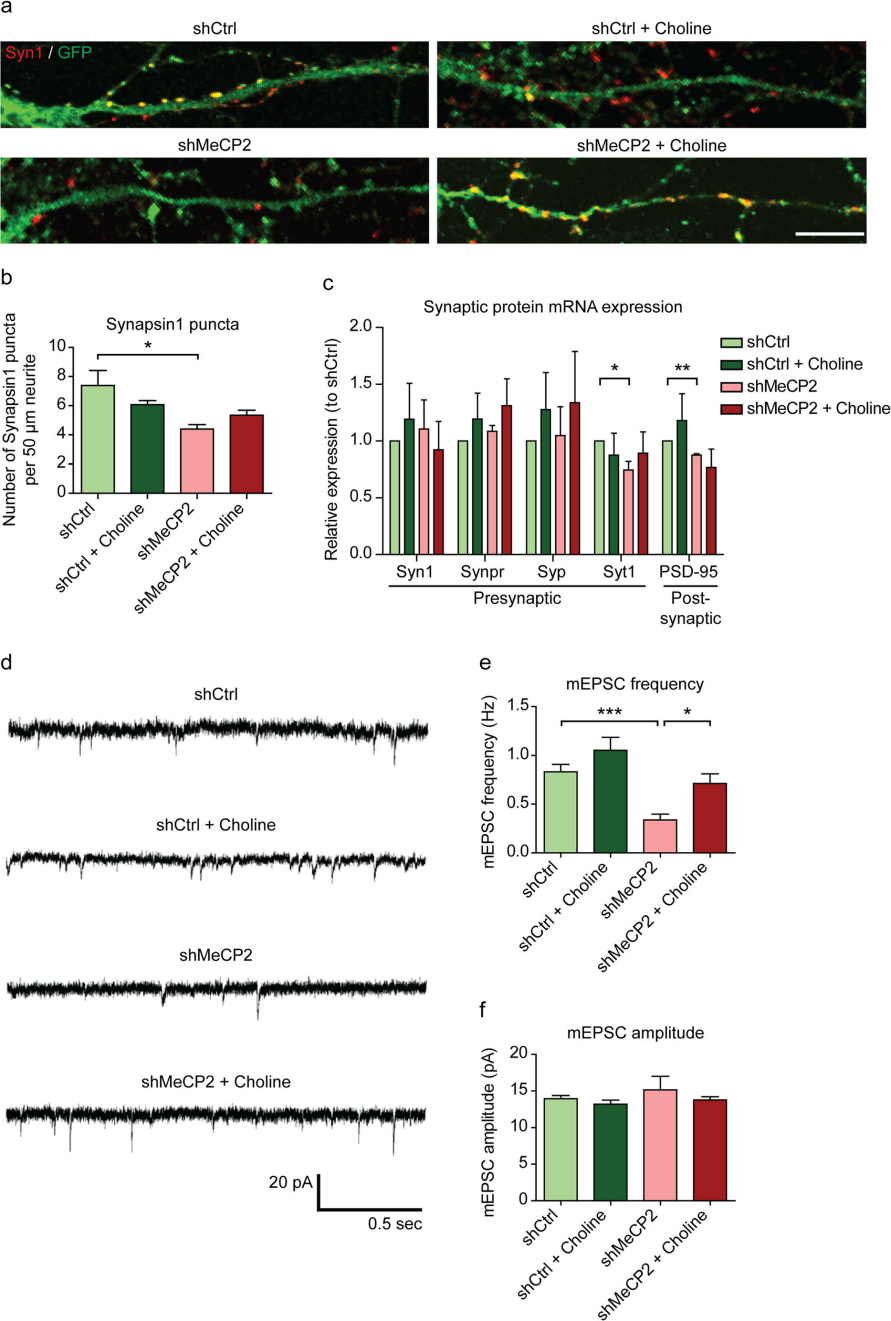
Choline supplementation restores synaptic defects in shMeCP2 neurons **a** Representative images of Synapsin1^+^ puncta (red) on GFP^+^ neurites (green) of DIV 14 cortical neurons. Co-localized puncta (yellow) were quantified. Scale bar represents 10 μm. **b** Quantification of Synapsin1^+^ puncta per 50 μm of neurite length. *n* = 3 cultures. **c** mRNA expression of selected presynaptic and postsynaptic proteins in shCtrl and shMeCP2 neuronal cultures with or without choline supplementation. Expression levels were normalized to the housekeeping gene Gapdh, and expressed relative to shCtrl levels. *n* = 5 cultures. Syn1, Synapsin1; Synpr, Synaptoporin; Syp, Synaptophysin; Syt1, Synaptotagmin1; PSD-95, Postsynaptic density 95. **d** Representative miniature excitatory postsynaptic current (mEPSC) recordings from shCtrl and shMeCP2 DIV 14 hippocampal neurons with or without choline supplementation. **e** and **f** Graphs show mean mEPSC frequency **e** and amplitude **f** of shCtrl and shMeCP2 neurons with or without choline supplementation. *n* = 19–35 neurons from six cultures. All values are presented as mean ± s.e.m. * *p* < 0.05, ** *p* < 0.01 and *** *p* < 0.001. One-way ANOVA with Bonferroni post-hoc

We next examined whether the enhancements in neuronal morphology led to subsequent improvements in neuronal functionality. Neurons were immunostained for the presynaptic protein synapsin1 (Syn1), and the number of signal puncta on neurites was counted. The lower number of Syn1^+^ puncta in shMecP2 neurons as compared to shCtrl neurons was increased upon choline supplementation (Fig. 4a, b). In addition, we found a trend of increased expression (approximately 10–30%) of *Synpr*, *Syp*, and *Syt1* mRNA in neurons treated with choline (Fig. 4c). Hence, choline supplementation seems to have an effect on the expression of synaptic proteins in shMeCP2 neurons.

To investigate the functional properties of shMeCP2 neurons with choline treatment, we performed whole-cell patch clamp recording. Miniature excitatory postsynaptic current (mEPSC) frequency was significantly lower in shMeCP2 neurons (0.34 ± 0.059 Hz) as compared to shCtrl neurons (0.83 ± 0.077 Hz) (Fig. 4d, e). In the choline-treated shMeCP2 neurons, mEPSC frequency significantly increased to 0.71 ± 0.099 Hz (Fig. 4d, e). The mEPSC amplitude of the neurons did not change significantly in the shMeCP2 neurons, nor with choline supplementation (Fig. 4d, f). Taken together, these results show that choline treatment to shMeCP2 neurons can ameliorate defects in synaptic transmission, possibly through increasing the number of functional connections.

### The Phosphatidylcholine Synthesis Pathway Is the More Essential Pathway Mediating the Rescue of Neurite Length in MeCP2-knockdown Neurons by Choline

**Fig. 5:**
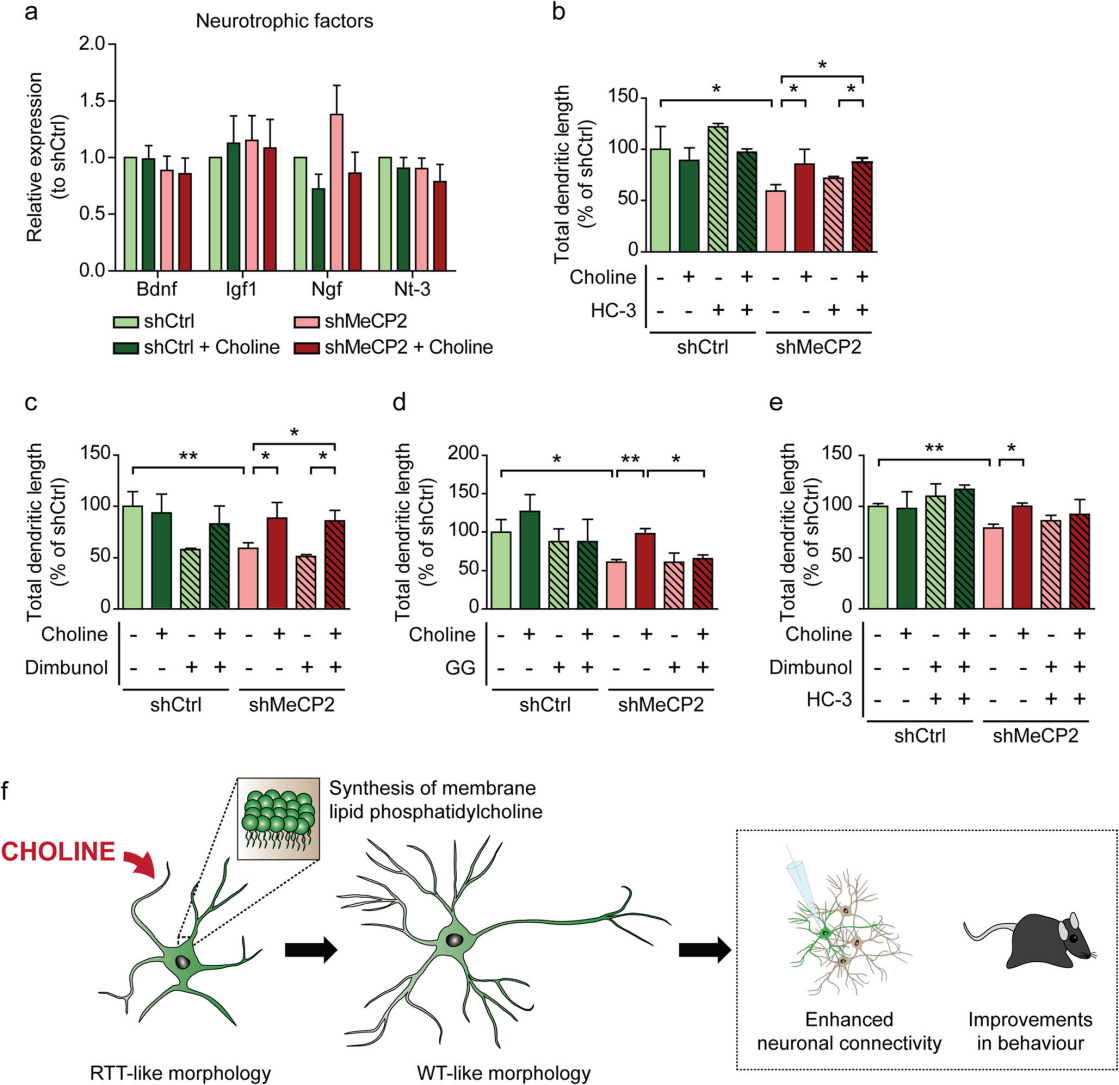
Possible mechanism of action of choline **a** mRNA expression of selected neurotrophic factors in shCtrl and shMeCP2 neuronal cultures with or without choline supplementation. Expression levels were normalized to the housekeeping gene Gapdh, and expressed relative to shCtrl levels. *n* = 5 cultures. Bdnf, Brain-derived neurotrophic factor; Igf1, Insulin-like growth factor 1; Ngf, Nerve growth factor; Nt-3, Neurotrophin 3. **b**–**e** Quantification of total dendritic length of shCtrl and shMeCP2 neurons with (+) or without (−) choline supplementation, expressed as a percentage of shCtrl levels. Neurons were cultured in the presence of specific inhibitors: 2 μM HC-3 to block acetylcholine synthesis **b**, 1.7 mM Dimbunol to block DNA methylation **c**, 5 μM GG to inhibit phosphatidylcholine synthesis **d**, and double inhibition with both Dimbunol and HC-3 **e**. *n* = 3 cultures. All values are presented as mean ± s.e.m. * *p* < 0.05 and ** *p* < 0.01. One-way ANOVA with Bonferroni post-hoc. **f** Illustration of the possible mechanism of choline in ameliorating RTT-like neuronal deficits and behaviour through its role in the synthesis of phosphatidylcholine

Finally, we wanted to elucidate the mechanistic pathway through which choline exerts its effects to bring about changes in the morphology of shMeCP2 neurons. Consistently across the panel of neurotrophic factors that we screened, we found no significant differences in their expression in the shMeCP2 neurons, or with choline supplementation (Fig. 5a). This indicates that the improvements in morphology of the choline-treated shMeCP2 neurons were not neurotrophic factor-induced.

It is possible that choline could be exerting its effects via one of its three main contributory roles in maintaining the cellular physiology of neurons. Choline is a precursor of the neurotransmitter acetylcholine, through accepting an acetyl group from acetyl-Co-enzyme A via choline acetyltransferase. It is also a key methyl group donor for methylation events, such as neuronal activity-induced DNA methylation of genes related to neuronal plasticity [31]. Finally, choline acts as a precursor of phosphatidylcholine, an essential lipid component of neuronal membranes. In order to determine which was important for improving neuronal morphology in shMeCP2 neurons, we blocked each of the pathways with specific inhibitors, and measured total dendritic length as a readout.

In shMeCP2 neurons cultured with choline and in the presence of HC-3, an inhibitor of acetylcholine synthesis [32], their mean total dendritic length was significantly greater than that of the untreated shMeCP2 cells with HC-3 added (Fig. 5b). Likewise, for shMeCP2 neurons treated with choline and in the presence of Dimbunol, an inhibitor of DNA methylation [33], their mean total dendritic length was significantly greater than that of the untreated shMeCP2 cells with Dimbunol added (Fig. 5c). On the other hand, when an inhibitor of phosphatidylcholine synthesis (GG) [34] was added to choline-supplemented shMeCP2 neurons, their mean total dendritic length was not significantly different from that of the untreated shMeCP2 cells (Fig. 5d). Further, when HC-3 and Dimbunol were used concurrently, there was a trend of increased total dendritic length in the shMeCP2 neurons with choline treatment as compared to those without (Fig. 5e). These results are strongly suggestive that the phosphatidylcholine synthesis pathway is essential for mediating the morphological, and possibly functional, changes seen in choline-supplemented shMeCP2 cortical neurons (Fig. 5f).

## Discussion

In recent years, there has been growing interest in the field of nutraceuticals, where nutritional components are used to augment, or in place of, traditional drug treatments in the therapy of certain diseases. The attractiveness of a nutraceutic approach lies in its “wholesomeness”, as the artificiality of synthetic drugs is done away with. Furthermore, if multiple treatments are needed, a less invasive intervention would be much preferred. This eases the process of translation to human studies. Nutritional therapy is already used in most cases of RTT to treat the nutritional deficiencies caused by feeding difficulties and gastrointestinal complications. Therefore, it would not be difficult to augment the diet plans of patients to ensure that they receive sufficient choline.

Choline is a water-soluble vitamin that is essential to the human diet, and it can be commonly found in most foods. The amount of choline ingested by the lactating dams in this study (13 mg/day, approximately 1.7 times the required daily intake of choline for their given weight) is well within the tolerable limits for adequate choline intake [35]. The paradigm used in this study for supplying choline to the postnatal pups is similar to those in previous studies of choline supplementation to RTT mouse models [18, 19]. Choline passes readily through the blood-brain barrier via the blood-brain barrier choline transporter, and into neurons mainly via the low affinity choline transporter and high affinity choline transporter systems [36–39]. The distribution and metabolism of choline in the brain are influenced by the choline content of the diet [40–42]. Increases in free choline and choline metabolites have been reported in the brains of rodents and humans following choline ingestion [40–44]. The magnitudes of the increases vary widely (approximately 25–400%), due to the different systems and administration protocols used, the latencies in measuring the choline-containing compounds, and the techniques used for measurement [43–47]. In an earlier study, Millington and Wurtman [44] administered a single dose of choline orally to rats at a similar concentration (20 mmol/kg) as that used in this study; they reported a 26% increase in choline-containing metabolites. Congruently, a 10–22% increase in choline metabolites was observed in human subjects given a single 50 mg/kg body weight oral dose of choline [43]. Hence, we conservatively estimate an increase of at least 20% in choline and choline-containing metabolites in the brains of our supplemented animals. It is acknowledged that the exact concentration of choline in the brains of the animals used in this study was not measured, and that the determination of such warrants future investigation.

To our knowledge, it is unclear whether RTT patients are deficient in choline. Some studies reported elevated levels of choline, while others found no difference in choline levels with respect to controls [48–53]. We showed here that choline’s rescue of RTT deficits may not be as straightforward as restoring cholinergic function as previously thought [18, 19]. We found that the phosphatidylcholine synthesis pathway was responsible for the improved neuronal morphology of shMeCP2 neurons. Phosphatidylcholine is implicated in having an impact on neuronal extension [54]. It is possible that the diminished morphology of RTT neurons is a result of dysregulation in the lipid synthesis pathway. This is in corroboration with our previous study, where differences in the levels of lipid metabolites of neurons derived from induced pluripotent stem cells from RTT patients and their isogenic controls were reported [55]. Separately, in vivo magnetic resonance imaging and spectroscopy of *Mecp2*^*−/y*^ mice revealed alterations in their profile of choline-containing phospholipids [56]. By narrowing it down to the phosphatidylcholine synthesis pathway, this study has clarified choline’s mechanism of action in RTT treatment. Examining the roles of various choline-containing lipid metabolites and enzymes involved in lipid metabolism in RTT pathogenesis and therapy would thus be intriguing.

Deficits in behaviour of RTT patients can be attributable to dysfunctional neurotransmission. To this end, we have shown functionally that choline supplementation re-established neuronal connectivity in vitro. Moreover, the effects of choline supplementation can be translated to behavioural improvements. We postulate that choline supplementation modulates the plasticity of RTT neurons, enabling them to form functional connections, leading to changes in behaviour. It should be noted that the improvements in motor coordination of animals supplemented with choline at either time points (early or late) were similar. This suggests that as long as the existing neuronal framework is available, an intervention that is able to affect its plasticity would be able to produce an effect; this property can be harnessed to treat RTT. Since the model that we have used here is a neuron-specific knockout, it is recognized that any possible effects of choline on the other cell types in the brains of the animals in this study were not examined. Astrocytes and microglia are able to take up and metabolize choline [57]. Choline-containing metabolites are important for astroglial signal transduction, differentiation, and regulation of apoptosis [58, 59]. They also have anti-inflammatory properties, leading to inhibition of microglia activation and modulation of cytokine levels [60–64]. Hence, supplementation of choline could potentially induce neuroprotective mechanisms in non-neuronal cells. Further studies need to be done to confirm this.

Although RTT primarily affects females, only male animals were used in the behavioural assays shown in this study. The males in mouse models of RTT are generally reported to have greater consistency in symptom presentation across animals [65–67], and their symptoms are typically more severe than those of the females [68–70]. It is, thus, more practical to use male animals as any aberrations due to the intervention can be readily assessed. The results gleaned here can then be extrapolated to female animals. It would be prudent to test the effects of choline supplementation in both sexes in future investigations.

To our knowledge, this is the first study showing mechanistic insights on choline in rescuing RTT deficits. Neuronal morphology and behaviour were shown here to be improved with choline supplementation. It is hoped that this study would help widen the field of nutraceuticals, so that more can be done to investigate their use in the treatment of RTT and other disorders. Further studies can also be conducted to examine the role of lipid signalling in RTT, so as to better understand its pathogenesis. The results gleaned from this study can provide further understanding on the mechanisms of the disorder, as well as open new avenues in discovering novel and more specific therapeutic targets for RTT.

## Experimental Procedures

### Primary Neuron Culture

Cortical and hippocampal primary neuronal cultures were obtained via dissociation of brain tissues from embryonic day 18 (E18) rat embryos. Detailed procedures for the dissociation and maintenance of the primary neuronal cultures can be found in [71]. Time-mated Sprague-Dawley dams used were euthanized in accordance with guidelines set out by the SingHealth Institutional Animal Care and Use Committee. Briefly, E18 embryos were extracted, and their brains were harvested and placed in ice-cold EBSS containing 10 mM HEPES. The cortices and hippocampi were dissected from the brain and digested with papain in separate preparations for 30 min at 37 °C. The digested tissues were then re-suspended in neuronal plating medium (Minimum Essential Medium containing 10% foetal bovine serum, 1 × N2 supplement, 3.6 mg/ml glucose, and 1 × penicillin/streptomycin). The tissue suspension was passed through a 70-μm cell strainer to sieve out tissue clumps. The tissue suspension was then passed through a 7.5% BSA/PBS layer by centrifuging at 200×*g* for 5 min. The resultant cell pellet was re-suspended in neuronal plating medium and seeded onto poly-l-lysine-coated glass coverslips or culture plates. The plating medium was exchanged for maintenance medium (neurobasal medium supplemented with 1 × B27 supplement, 0.5 × l-glutamine, and 1 × penicillin/streptomycin) the next day. The cells were maintained in a humidified incubator at 37 °C and 5% CO2 level until the required time points. A third of the culture medium was exchanged for fresh medium every 7 days.

### Choline Supplementation and Addition of Inhibitor Compounds to Neuronal Cultures

Choline chloride [(2-hydroxyethyl)trimethylammonium chloride] (Sigma-Aldrich) was dissolved in sterile water and used at a working concentration of 100 μM. The neuronal cultures were supplemented with choline every 2 days, starting from days in vitro 4 (DIV 4) until the end of their culture periods.

Hemicholinium-3 (HC-3) (Sigma-Aldrich) was dissolved in sterile water and used at a working concentration of 2 μM. 3,3-dimethyl-1-butanol (Dimbunol) (Sigma-Aldrich) was diluted in cell culture medium to a working concentration of 1.7 mM. Geranylgeraniol (GG) (Sigma-Aldrich) was diluted in cell culture medium and used at a working concentration of 5 μM. The inhibitor compounds were added to neuronal cultures at the same time as the choline supplementation.

### shRNA Vector Production and Transduction

Lentiviruses for transduction were produced by transfection into HEK293 cells via calcium phosphate precipitation, and collection via ultracentrifugation, as previously described in [72]. The sequences for the control shRNA (shCtrl) and that for knocking down the expression of MeCP2 (shMeCP2) are as follow: AGTTCCAGTACGGCTCCAA (shCtrl), and GGGAAACTTCTCGTCAAGA (shMeCP2) [73]. Viruses were added to the neuronal cultures at DIV 1.

### Animals and Choline Supplementation In vivo

All procedures were approved by the SingHealth Institutional Animal Care and Use Committee. *Mecp2*-floxed (B6.129P2-Mecp2^tm1Bird^/J) and *CamKIIα-cre* (B6.Cg-Tg(Camk2a-cre)T29-1Stl/J) mice were purchased from Jackson Laboratories. The males were removed from the breeding cages near the end of the gestation period. The subsequent individually-housed dams were kept with their delivered pups until weaning at postnatal day 21 (P21). Weaned pups were housed in cages with up to five same-sex littermates, with food and water provided ad libitum. All animals were maintained on a 12-h light/dark cycle, with lights on at 0700 h and lights off at 1900 h.

Lactating dams were provided with drinking water supplemented with 25 mM choline and 50 mM saccharin, or with 50 mM saccharin only (vehicle). There were two experimental supplementation periods: from the day of birth of the litter (P1) until weaning (P21) (early supplementation), and from 6 weeks to 9 weeks of age (late supplementation).

### Behavioural Testing

All behavioural tests were carried out in a dedicated experiment room. Only male animals were used. All behavioural analyses were performed blinded. Animals were put into the experiment room at least 4 h before testing for acclimatization. Animals on the early supplementation regime were tested when they were between 6 to 8 weeks old, while those on the late supplementation regime were tested between 12 to 14 weeks of age.

### Open Field Assay

Mice were placed in a 40 × 40 × 40 cm transparent Plexiglas chamber and allowed to explore freely for 60 min. A VersaMax Animal Activity Monitoring System (AccuScan Instruments, Inc.) was used to record activity, and its accompanying VersaDat software (AccuScan Instruments, Inc.) was used for data generation. Total distance traveled by the animals was taken as the total distance moved by the animals in the arena over the test period of 60 min. Exploratory behaviour in a novel environment was taken as total distance traveled in the first 5 min of being placed in the arena. For analysis of anxiety-like behaviour, the arena was divided into the perimeter “margins” region (area prescribed by 10 cm away from the edges) and the middle “centre” region (20 × 20 cm).

### Rotarod Assay

To measure the animals’ motor coordination, an accelerating (from 4 to 40 rpm over 4 min) rotarod (San Diego Instruments) was used. Animals were tested over a period of four consecutive days, with one test session each day. Each test session consisted of four trials, with animals rested for a minimum of 10 min in its home cage between each trial. Time spent on the rotarod was recorded by an automated system which stops when the animal falls and breaks the photobeam.

### Crawley Box

Animals were placed in a three-chambered setup, with a juvenile stranger mouse (one that the subject has had no prior interaction with) in one of the side chambers, and an inanimate object in the other side chamber. The social preference index is given by the ratio of the amount of time that the subject animal spent interacting with the stranger animal to the total amount of time spent interacting with both the stranger animal and the object.

### Immunohistochemistry

Cells were washed with PBS and then fixed with 4% paraformaldehyde (with 4% sucrose) for 15 min at room temperature. The cells were washed twice (5 min each wash) with Tris-buffered saline (TBS) and permeablized with 0.1% Triton X-100 in TBS (TBS-Tx) for 5 min. They were blocked with 5% donkey serum in TBS-Tx for 2 h at room temperature. They were then incubated with the primary antibodies overnight at 4 °C. Primary antibodies used were: anti-GFP (1:3000, Rockland), anti-MAP2 (1:1000, Sigma), and anti-Synapsin1 (1:500, Abcam). After three washes with TBS-Tx, the cells were incubated with secondary Alexa Fluor® antibodies (1:500, Thermo Fisher Scientific) for 2 h at room temperature. The cells were stained with DAPI (1:5000) for 10 min at room temperature before they were mounted onto glass microscope slides and allowed to dry before imaging.

### Golgi Staining

Golgi staining was performed using the FD Rapid GolgiStain™ Kit (FD NeuroTechnologies Inc.), with slight modifications to the manufacturer’s instructions. Animals were anesthetized and perfused trans-cardially with ice-cold saline. Their brains were harvested and immersed in the impregnation solution and stored in the dark at room temperature for 5 days. They were then transferred to 30% sucrose solution for cryo-protection for 24 h. The brains were subsequently immersed in the fixation solution and kept in the dark for a week.

The brains were cut on a cryostat at −22 to −24 °C. Sections of 150 μm were obtained and mounted onto gelatin-coated glass microscope slides and left to air-dry. The dried sections were then washed with double-distilled water and immersed in the staining solution for 10 min. After which, the sections were dehydrated serially in increasing concentrations of ethanol—50%, 75%, 95%, and absolute. A clearing step in xylene was performed before the sections were mounted with DPX Mountant (Sigma-Aldrich).

### Confocal Microscopy and Image Analysis

Images were taken using a Zeiss LSM 710 confocal microscope, and analyzed with ImageJ and LSM Image Browser. Soma size, total dendritic length, number of branches, and number of Syn1^+^ puncta were measured and counted manually. Sholl analysis was performed with images of traced dendritic arbors using an ImageJ plugin [74]. Sampling step size was set at 10 μm. Imaging of Golgi-stained sections was conducted using the T-PMT function on the Zen software. Thirty to 40 cells from each condition per culture batch or animal were analyzed.

### Quantitative Real-time Polymerase Chain Reaction

Total RNA was isolated with Trizol® according to the manufacturer’s instructions. cDNA was synthesized from 1 μg of RNA using iScript™ Select cDNA Synthesis Kit (Bio-Rad). Quantitative PCR was performed using iQ™ SYBR® Green Supermix (Bio-Rad) on a CFX96™ Real-Time System (Bio-Rad). Sequences of primers used are as follows—Bdnf: ATAGGAGACCCTCCGCAACT (forward [F]), CTGCCATGCATGAAACACTT (reverse [R]); Gapdh: CATCACTGCCACTCAGAAGA [F], CAACGGATACATTGGGGGTA [R]; Igf1: ACGCTCTTCAGTTCGTGTGT [F], TCAGCGGAGCACAGTACATC [R]; Ngf: ACAGGCAGAACCGTACACAG [F], CTATTGGTTCAGCAGGGGCA [R]; Nt-3: CCGACAAGTCCTCAGCCATT [F], TTGCGACGTTTTGCACTGAG [R]; Syn1: CAGTTTGGTCATTGGGCTGC [F], TCGAACCATCTGGGCAAACA [R]; Synpr: ATCAAGGCGGCTACAACCAA [F], CATCAGAAGTTGGCCCCAGA [R]; Syp: TGCCATCTTCGCCTTTGCTA [F], TTGGTAGTGCCCCCTTTGAC [R]; Syt1: GAAGATGGATGTGGGTGGCT [F], AGCATGTCTGACCAGTGTCG [R]; and PSD-95: GCCAATTCTCCCCCTGTGAT [F], CCCCCTCTGTTCCATTCACC [R]. Melt curves were manually inspected to ensure primer specificity. PCR product sizes were also checked via agarose gel electrophoresis.

### Western Blotting

Proteins were separated on 10% SDS-PAGE gels under reducing conditions. The proteins were then transferred onto polyvinyldifluoride membranes and blocked with 5% BSA in TBS with 0.1% Tween 20 (TBS-Tween) for 1 h at room temperature. The membranes were blotted with the desired primary antibodies and incubated overnight at 4 °C. Primary antibodies used were: anti-MeCP2 (1:1000; Cell Signalling), and anti-Tubulin (1:20000; Sigma). After washing thrice with TBS-Tween (10 min each wash at room temperature), the membranes were then blotted with secondary antibody-horseradish peroxidase conjugates (GE Healthcare) for 1 h at room temperature. SuperSignal West Pico and Femto Chemiluminescent Substrate (Thermo Fisher Scientific) was used for developing the blots, and a GE LAS4000 imaging machine was used to visualize them. Band densitometry analysis was done using ImageJ.

### Electrophysiology

DIV 14 hippocampal neurons were recorded using whole-cell patch clamp. Cells were visualized using DIC optics (Olympus) and patched with pipettes (2–4 MΩ) fashioned from borosilicate glass capillaries (G150F-3, Warner Instruments) using a vertical puller (PC-10, Narishige). The internal solution for the patch pipette contained (in mM): 120 K-gluconate, 9 KCl, 10 KOH, 3.5 MgCl_2_, 4 NaCl, 10 HEPES, 4 Na_2_ATP, 0.4 Na_3_GTP, 17.5 sucrose, and 0.5 EGTA. Neurons were bathed in an external solution containing (in mM): 125 NaCl, 24 NaHCO_3_, 10 glucose, 2.5 KCl, 1 NaH_2_PO_4_, 2.5 CaCl_2_, 2 MgCl_2_, 0.001 tetrodotoxin (TTX), and 0.02 bicuculline methiodide (BMI). TTX and BMI were acquired from Tocris Bioscience. Recordings were acquired using a patch clamp amplifier (Axon Instruments), and the signals were digitized and sampled at 10 kHz, filtered at 2 kHz, and analyzed using pClamp 10.3 software (Axon Instruments). Neurons were held at − 70 mV after whole-cell formation and series resistance was checked before and after recording. Data in which series resistance changed more than 20% were discarded. mEPSC amplitude and frequency were analyzed with MiniAnalysis software (Synaptosoft).

### Statistical Analysis

Statistical analyses were performed with GraphPad PRISM. The statistical tests used are stated in the figure legends. Statistical significance was set at *p* < 0.05.
